# Tafazzin Knockdown in Mice Leads to a Developmental Cardiomyopathy With Early Diastolic Dysfunction Preceding Myocardial Noncompaction

**DOI:** 10.1161/JAHA.111.000455

**Published:** 2012-04-24

**Authors:** Colin K. L. Phoon, Devrim Acehan, Michael Schlame, David L. Stokes, Irit Edelman-Novemsky, Dawen Yu, Yang Xu, Nitya Viswanathan, Mindong Ren

**Affiliations:** Department of Pediatrics (Pediatric Cardiology), New York University School of Medicine, New York (C.K.L.P., N.V.); Department of Cell Biology, New York University School of Medicine, New York (D.A., M.S., D.L.S., I.E.-N., D.Y., M.R); Department of Anesthesiology, New York University School of Medicine, New York (M.S., Y.X.); Skirball Institute of Biomolecular Medicine, New York University School of Medicine, New York (D.A., D.L.S.); New York Structural Biology Center, New York (D.L.S.)

**Keywords:** Barth syndrome, cardiolipin, mitochondrial disease, noncompaction cardiomyopathy, tafazzin

## Abstract

**Background:**

Barth syndrome is a rare, multisystem disorder caused by mutations in *tafazzin* that lead to cardiolipin deficiency and mitochondrial abnormalities. Patients most commonly develop an early-onset cardiomyopathy in infancy or fetal life.

**Methods and Results:**

Knockdown of *tafazzin* (TAZKD) in a mouse model was induced from the start of gestation via a doxycycline-inducible shRNA transgenic approach. All liveborn TAZKD mice died within the neonatal period, and in vivo echocardiography revealed prenatal loss of TAZKD embryos at E12.5-14.5. TAZKD E13.5 embryos and newborn mice demonstrated significant *tafazzin* knockdown, and mass spectrometry analysis of hearts revealed abnormal cardiolipin profiles typical of Barth syndrome. Electron microscopy of TAZKD hearts demonstrated ultrastructural abnormalities in mitochondria at both E13.5 and newborn stages. Newborn TAZKD mice exhibited a significant reduction in total mitochondrial area, smaller size of individual mitochondria, reduced cristae density, and disruption of the normal parallel orientation between mitochondria and sarcomeres. Echocardiography of E13.5 and newborn TAZKD mice showed good systolic function, but early diastolic dysfunction was evident from an abnormal flow pattern in the dorsal aorta. Strikingly, histology of E13.5 and newborn TAZKD hearts showed myocardial thinning, hypertrabeculation and noncompaction, and defective ventricular septation. Altered cellular proliferation occurring within a narrow developmental window accompanied the myocardial hypertrabeculation-noncompaction.

**Conclusions:**

In this murine model, tafazzin deficiency leads to a unique developmental cardiomyopathy characterized by ventricular myocardial hypertrabeculation-noncompaction and early lethality. A central role of cardiolipin and mitochondrial functioning is strongly implicated in cardiomyocyte differentiation and myocardial patterning required for heart development. **(*J Am Heart Assoc*. 2012;1:jah3-e000455 doi: 10.1161/JAHA.111.000455.)**

## Introduction

Barth syndrome (MIM 302060) is a rare X-linked, multisystem disorder characterized by cardiomyopathy, skeletal myopathy, neutropenia, and growth retardation.^1–3^ The most common presentation is early cardiomyopathy, often occurring in infancy. Indeed, prenatal loss and/or morbidity can occur from fetal cardiomyopathy.^4^ Patients may exhibit a dilated or hypertrophic cardiomyopathy, endomyocardial fibroelastosis, and/or a developmental cardiomyopathy known as myocardial (left ventricular) noncompaction or hypertrabeculation (“spongy myocardium”). This last cardiomyopathy is considered a hallmark of Barth syndrome, and is also notable in other mitochondrial disorders.^5^

Barth syndrome is caused by mutations in *tafazzin*, which lead to severe deficiency and altered molecular species of the phospholipid cardiolipin.^6,7^ Tafazzin catalyzes cardiolipin remodeling reactions at the final stage of cardiolipin biosynthesis, producing so-called “mature” cardiolipin.^8^ In tafazzin-deficient cells, cardiolipin levels are lower, while monolysocardiolipin levels are markedly elevated, and the acyl chain composition is shifted toward less unsaturated species.^9,10^ For reasons not completely understood, normal cardiolipin profiles seem to be critical to the structural integrity and the bioenergetic function of mitochondria.^9,10^

Recently, a short-hairpin RNA (shRNA) -inducible tafazzin-knockdown (TAZKD) mouse model became available that exhibited an adult-onset cardiomyopathy associated with abnormal cardiolipin profiles and mitochondrial structural abnormalities.^11,12^ Using a modified induction scheme in the same TAZKD mouse, we found near-universal pre- and perinatal lethality. This early death was caused by a developmental cardiomyopathy, a phenotype that strikingly resembles the noncompaction cardiomyopathy of Barth syndrome. Our results support an important role for cardiolipin in myocardial patterning during heart development.

## Methods

### Animals and Doxycycline Transgene Induction

**Figure 1.**
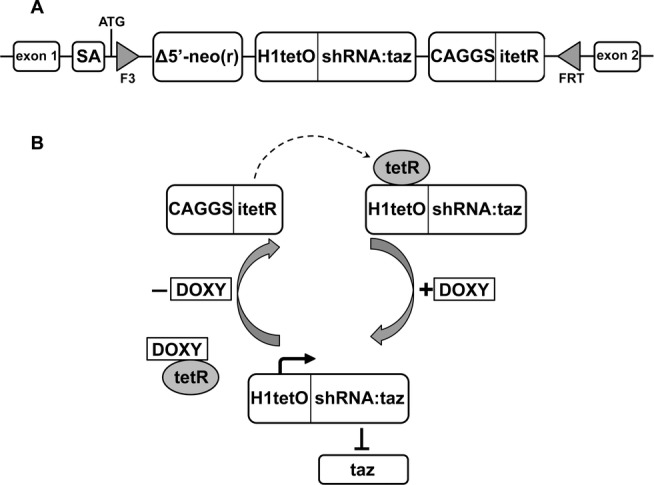
Schematic of *taz*-specific inducible shRNA-mediated targeting strategy. Doxycycline dose was 2 mg/mL in the drinking water (13). (A) Inserted by recombinase-mediated cassette exchange, the F3-FRT cassette contains the *taz*-specific shRNA H1-tet-On promoter, a tet repressor (tetR), and a neomycin selection gene; (B) the tetO-shRNA promoter is inactivated by tetR until doxycycline is added to the system, inactivating tetR, and allowing transcription of *taz*-specific shRNA; the system is reversible. Of several correctly targeted embryonic stem cell clones generated at TaconicArtemis, GmbH, one construct resulted in acceptable *taz* knockdown, and breeder mice were sent to investigators on request.

All protocols were approved by the Institutional Animal Care and Use Committee of NYU School of Medicine and Langone Medical Center, and conform to the Guide for the Care and Use of Laboratory Animals published by the National Institutes of Health (NIH). Mice were housed under temperature-controlled conditions under a 12-hour light/dark cycle with free access to drinking water and food. Transgenic mice were generated at TaconicArtemis, GmbH (Köln, Germany) under contract from the Barth Syndrome Foundation, and made freely available to investigators; these mice are now available through The Jackson Laboratory (Bar Harbor, ME). These TAZKD mice have been previously described in detail,^11,12^ on the basis of a tight inducible shRNA expression system^13^ (Figure 1). TAZKD transgenic mice used in this study are the heterozygote off-spring of heterozygote (male)-wildtype (female) C57Bl/6 crosses, as per TaconicArtemis’ protocol. The day after overnight mating that a plug was found was designated as E0.5. In brief, fresh solutions containing 2 mg/mL doxycycline (Frontier Scientific D-10056) and 10% sucrose were prepared every other day and kept dark. (The use of 10% sucrose was necessary to improve palatability of the doxycycline solution; so far as we know, such a diet has not been shown to result in abnormal myocardial patterning.) Doxycycline was administered to wildtype female mice on a continuous basis, to make sure doxycycline remained in the maternal system and the embryonic transgene would therefore be induced throughout gestation. However, because the males carry the transgene as heterozygotes, we fed the mating pairs regular water without doxycycline to reduce the possibility of male sterility, which is a known adverse effect in a Drosophila model of tafazzin deficiency.^14^ Doxycycline was reintroduced into the drinking water of the females on the day following overnight mating (day of plug).

We used the same induction protocol as in the original protocol,^13^ but doxycycline dosing differed from recent investigations (625 mg/kg chow 11; 200 mg/kg chow ^12^). We estimate our TAZKD mice ingested 3 to 10 times the doxycycline used in studies by Acehan et al^11^ and Soustek et al,^12^ because mice ingest on average 15 g/100 g body weight in food and 15 mL/100 g body weight water daily (http://www.lvma.org/mouse.html). To determine the kinetics of *tafazzin* knockdown and effects on development, doxycycline induction was initiated on different gestational ages as described later.

### Genotyping and Quantitative Reverse Transcription Polymerase Chain Reaction

Mice were genotyped by standard polymerase chain reaction using proteinase K digested tail or yolk sacs of mouse embryos. Real-time quantitative reverse transcription polymerase chain reaction was done in standard fashion.^15^ Briefly, this set of primers identified the TAZ-shRNA (where TAZ is *tafazzin*) transgene, and amplifies a 381 Bp fragment: 5′-CCATGGAATTCGAACGCTGACGTC-3′/5′-TATGGGCTATGAACTAATGACCC-3′. For quantitative reverse transcription polymerase chain reaction, the sequences of the *taz* primers used were as follows: (forward) 5′-GCAGACATCTGCTTCACCAA-3′/(reverse) 5′-TGAAGTCCATCCCTTTCTGG-3′. Briefly, total RNA was extracted using the acid-phenol guanidinium method (TriReagent, Sigma). Embryonic and newborn tissues were dissected quickly in iced phosphate-buffered saline and snap-frozen. RNA purification was performed using Ambion's TRIzol Plus RNA Purification Kit according to the manufacturer's instructions (Invitrogen). Total RNA was reverse-transcribed in standard fashion using Roche's Transcriptor First Strand cDNA Synthesis Kit (Roche Applied Science). Polymerase chain reaction was performed in 384-well format, and reactions were performed using an ABI Prism 7900HT Sequence Detection System (Perkin-Elmer Applied Biosystems) available through the NYU Cancer Institute Genomics Facility. To quantitate gene expression, we used the widely used ΔΔCt method, using SDS software (version 2.3, Applied Biosystems); the Ct data of a target transcript were expressed as relative changes between the two experimental conditions, measured within the exponential phase of the polymerase chain reaction. A correction was performed using a passive reference dye (Rox), which is present in the polymerase chain reaction master mix. Reactions with a Ct value >36 or with any evidence of nonspecificity (low melting temperatures or multiple peaks in melting point analysis) were excluded from the analysis. We calculated target gene expression relative to that of a subset of appropriate reference genes (β-actin, GAPDH, histone-3, ribosomal protein S6, and hydroxymethylbilane synthase), using the Bestkeeper algorithm.^15^

### Cardiolipin Analysis

Mouse tissues were homogenized and the homogenates were transferred immediately to glass tubes containing 2 mL methanol and 1 mL chloroform. These tubes were incubated at 37°C for 30 minutes. Lipids were extracted according to Bligh and Dyer,^16^ extracts were dried under a stream of nitrogen and finally resuspended in 100 μL chloroform/methanol (1:1). For lipid mass spectrometry, we followed the method of Sun et al^17^ with minor modifications. An aliquot of the extract was diluted 1:10 in 2-propanol/acetonitrile (3:2). A total of 10 μL of the diluted solution were mixed with 10 μL of matrix solution, containing 9-aminoacridine (10 g/L) in 2-propanol/acetonitrile (3:2). One microliter of this mixture was applied to a sample well and was allowed to dry. Mass spectrometry was performed with the MALDI micro-MX instrument from Waters operated in negative ion mode and in reflectron mode. The instrument was calibrated daily with a reference mixture of myristoyl-lyso-phosphatidylglycerol (*m*/*z*=455.2415), dioleoyl-phosphatidylglycerol (*m*/*z*=773.5338), dilinoleoyl-phosphatidylglycerol (*m*/*z*=738.5079), and tetralinoleoyl-cardiolipin (*m*/*z*=1447.9650). Voltages were set to the following parameters: pulse voltage 2000 V, detector voltage 2200 V, flight tube voltage 12 kV, reflectron voltage 5.2 kV, and negative anode voltage 3.5 kV. Time-lag focusing delay was set to 700 ns. The nitrogen laser (337 nm) was fired at a rate of 5 Hz and 10 laser shots were acquired per subspectrum. We typically acquired 100 to 200 subspectra per sample in a mass range from 400 to 2000 Da. Data were analyzed with the software MassLynx 4.1.

### Prenatal and Postnatal Mouse Echocardiography

For noninvasive imaging, adult mice (including pregnant mice) were anesthetized with isoflurane via nose cone (maintenance: 1% isoflurane mixed with 100% medical oxygen at flow rate 2.5 L/min), with strict thermoregulation (37±1°C) to optimize physiological conditions and reduce hemodynamic variability.^18,19^ Neonatal and very young juvenile mice (<4 weeks of age), which were too small or fragile for nosecone anesthetic, were held very gently with one hand (permitting self-thermoregulation); the mice remained very calm and still, with no signs to suggest that they experienced any significant discomfort, distress, or pain. In vivo transthoracic imaging for postnatal mice and transabdominal imaging for timed-pregnant mice was used, permitting accurate localization of embryos and as detailed by our laboratory previously.^18–20^ Two-dimensional, spectral Doppler, and color Doppler flow echocardiography was performed using the Vevo 2100 (Visual Sonics, Toronto, ON, Canada) with a 40 MHz center frequency transducer. To minimize bias, we performed all echocardiographic measurements prior to knowledge of genotype.

### Electron Microscopy

Electron micrographic analyses have been previously described,^21^ and detailed mitochondrial morphometrics were performed. Briefly, heart tissue was preserved by conventional chemical fixation, followed by gradual ethanol dehydration, infiltration, and curing with epoxy resin, and staining with uranylacetate and Sato Lead Stain. The sample blocks were sectioned to 50 to 70 μm thickness. The samples were imaged using a Philips CM12 microscope operated at 120 kV. The following measurements were performed using Image J (NIH): (1) mitochondrial area density (total cross sectional area of mitochondria divided by total cytosol area); (2) mitochondrial size (average cross-sectional area of mitochondria); (3) mitochondrial number (number of mitochondria per cell area, excluding nucleus); (4) cristae density (number of cristae per mitochondrial area); (5) mitochondrial orientation to myofibrillar structures (A, angle between long axes of mitochondria and associated myofibril bundle, measured as [90 minus {the angle between the *Z*-disk and the long axis of the adjacent associated mitochondrion}]; B, distance between the near edge of the myofibril bundle and the center of the adjacent associated mitochondrion). From multiple animals in each experimental group, multiple measurements from each of multiple random sections from cardiomyocytes were analyzed for each animal.

### Histology and Immunohistochemistry

We used standard histological and immunohistochemical methods to determine cellular morphometry, degree of myocardial fibrosis, myocardial cellular proliferation rates, and apoptosis. Standard 6 μm paraffin histological sections were used to determine primarily the presence of myocardial hypertrabeculation. Sections were stained with hematoxylin and eosin for standard morphometric analysis, and Masson's trichrome stain to gauge fibrosis in cardiomyopathic hearts. To determine the degree of myocardial noncompaction/hypertrabeculation, we adapted the protocol of Ishiwata et al^22^ to determine the degree of hypertrabeculation in the left ventricular lateral wall, left ventricular apex, and right ventricular lateral wall. Random samplings of left ventricular free wall and adjacent intracavitary trabeculations were measured using Image J, and the ratios of trabecular area to left ventricular free wall compact zone area were calculated in the left ventricular lateral wall, left ventricular apex, and right ventricular lateral wall of newborn pups. We quantified total cell proliferation in the heart by Ki-67 immunochemistry. Although many myocardial cells, including cardiomyocytes, are not yet fully differentiated at the stages we are studying,^23^ cardiomyocytes comprise the great majority of cells in the developing heart.^24^ Caspase-3 assays were utilized to determine apoptosis in the ventricular myocardium. Positively labeled cells were counted against blue-staining nuclei from several high-power fields, from both the trabecular and the compact myocardium separately.

### Statistical Analysis

Data are expressed as mean±SEM. Differences among groups were analyzed using the Mann-Whitney *U* test for continuous variables and Fisher's exact test for categorical variables. Statistical significance was set at *P*<0.05.

## Results

### *Tafazzin* Knockdown Leads to Prenatal and Perinatal Lethality

**Figure 2.**
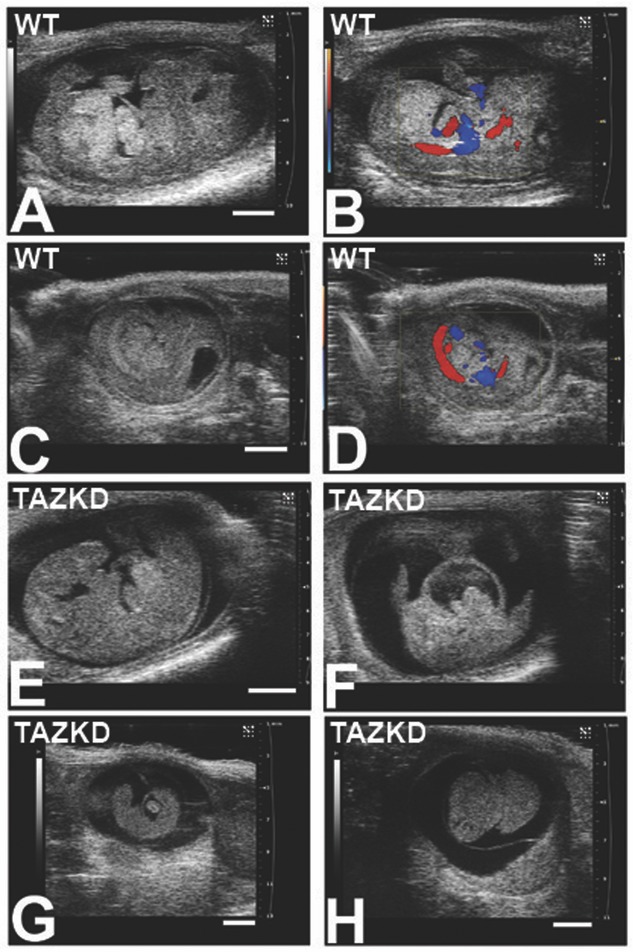
Doxycycline-inducible shRNA-mediated *tafazzin* knockdown leads to prenatal lethality. In vivo echocardiography shows a spectrum of prenatal loss, and provides rapid morphological phenotyping of live and dead mouse embryos. Sagittal views demonstrate control wildtype (WT) E14.5 mouse embryo, B-mode/2-dimensional imaging (A) and color Doppler blood flow imaging (B); and WT E12.5 mouse embryos, sagittal views (C, D), to show differences in size with E14.5. A dead E14.5 TAZKD mouse embryo (E) demonstrates relatively well-preserved morphology indicating recent death; B-mode and color Doppler imaging showed lack of a heartbeat and blood flow. A transverse view of the chest in the same E14.5 embryo (F) better demonstrates the large pericardial effusion. In early stages of resorption (G), a dead E13.5 TAZKD embryo demonstrates a large pericardial effusion; the small size of the embryo indicates death at least 1 day before imaging. A littermate of (G), a dead E13.5 TAZKD embryo (H) shows further necrosis as indicated by homogeneous gray tissue, closer to complete resorption. Live TAZKD embryos at any stage were indistinguishable from WT live embryos by 2D echo imaging. Scale bars: 2 mm. TAZKD indicates *tafazzin* knockdown embryos.

We observed a significantly lower number of TAZKD pups born, which had not been reported previously.^11,12^ Therefore, we focused on in vivo echocardiography at various stages of embryonic development (E12.5, E13.5, E14.5), which showed varying numbers of TAZKD mice dead (Figure 2, Table 1). Furthermore, when we attempted to permit several litters to survive (without tissue harvest) in order to grow several TAZKD mice into adulthood, there were no TAZKD survivors beyond the first week of life. While some TAZKD pups indeed were found dead shortly after birth, others may have been cannibalized by their mothers because of their condition.

**Table 1.** Spectrum of Lethality Starting by E12.5

Stage	Total	WT Alive	WT Dead	TAZKD Alive	TAZKD Dead	*P*-value (Fisher's)
E12.5	14	7	1	3	3	0.245

E13.5	67	31	2	29	5	0.428

E14.5	28	18	1	3	6	0.001

Newborn	60	35	0	13	12	<0.0001

TAZKD indicates *tafazzin* knockdown (induced); WT, wildtype.

Mice were harvested at the stages shown in the Table; newborn mice were harvested within hours of birth. Fisher's exact test (two-tailed) was performed on 2×2 contingency tables of WT versus TAZKD mice, alive versus dead, and shows loss of expected Mendelian ratios of living TAZKD mice by E14.5. Additional data from longitudinal echocardiographic imaging of three litters further attested to prenatal and perinatal lethality: a total of 24 embryos (18 alive, six dead [nonresorbed]) were demonstrable at E12.5, but only 10 were born; eight lived (all WT) while two died early on the day of birth (both TAZKD).

### *Tafazzin* Knockdown Leads to Alterations in Cardiolipin and Mitochondrial Ultrastructure

**Figure 3.**
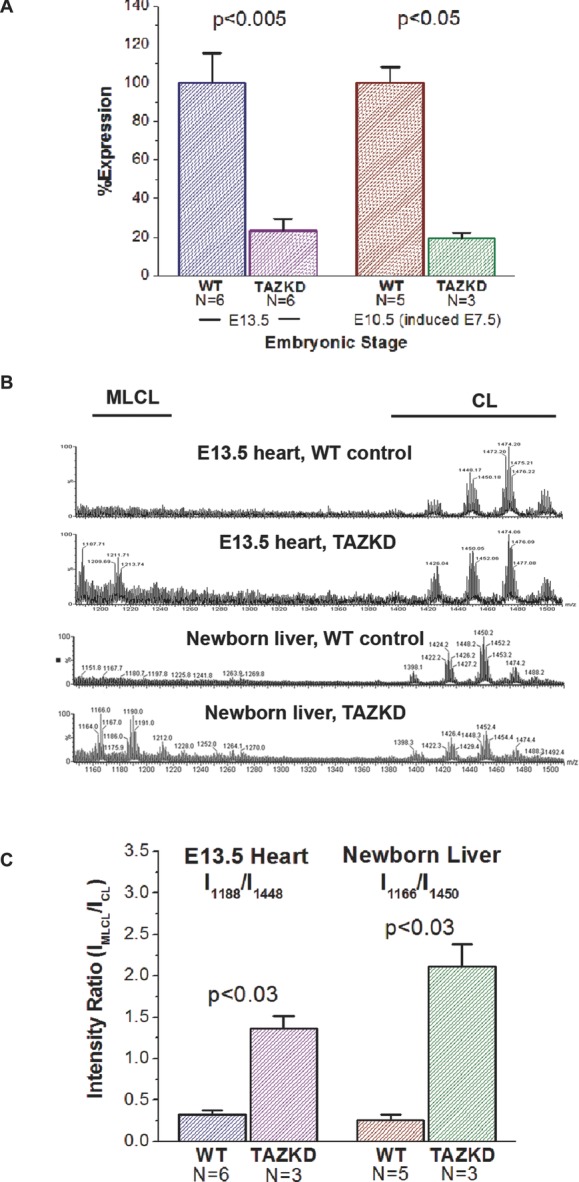
Effective *tafazzin* knockdown leads to altered cardiolipin profiles during development in TAZKD mice. Quantitative RT-PCR analysis of TAZKD mouse embryos shows *tafazzin* can be knocked down effectively during development (A). Induction from the start of gestation leads to approximately ∼80% reduction in taz mRNA by E13.5 ([A], left-hand bars). In addition, 3 days' induction (starting at E7.5) with doxycycline leads to the same degree of taz knockdown by E10.5 ([A], right-hand bars]. MALDI mass spectrometry [B] shows altered cardiolipin spectra in TAZKD E13.5 embryonic mouse hearts and newborn mouse livers; TAZKD tissues show a characteristic increase in the ratio of monolysocardiolipin species (MLCL) to cardiolipin (CL), also demonstrated by quantitative analysis of cardiolipin profiles (C). In E13.5 heart, ratios were obtained from molecular species at m/z 1188 (MLCL):1448 (CL), and in newborn liver, at 1166 (MLCL):1450 (CL). TAZKD indicates *tafazzin* knockdown mice; RT-PCR, reverse transcription polymerase chain reaction.

Effective *tafazzin* knockdown (>70% to 80%) was confirmed in E13.5 embryos and in newborns (Figure 3). Similar to other reports, TAZKD mice exhibited abnormalities in cardiolipin, but ours is the first report of abnormalities during embryonic development. In both TAZKD embryonic and newborn mice, the composition of cardiolipin molecular species was altered with a shift to a higher monolysocardiolipin:cardiolipin ratio (Figure 2); this increased MLCL:CL ratio is diagnostic for Barth syndrome.^9^

**Figure 4.**
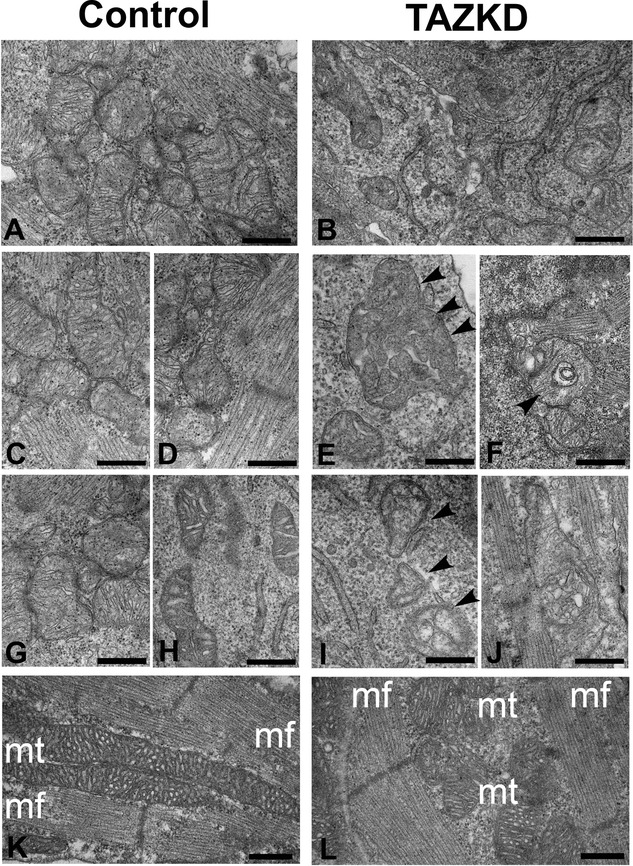
Electron micrographic analysis shows a spectrum of mitochondrial abnormalities similar to human Barth syndrome. We compared E13.5 embryonic wildtype (A, C, D, G, H) and TAZKD (B, E, F, I, J) cardiomyocytes, focusing on the mitochondria and regions around the mitochondria. Examples of abnormal mitochondrial ultrastructure include reduced mitochondrial density (A, B); abnormal cristae morphology (B, E, F, I, J), including disruption of cristae (B, E) and decreased cristae matrix density (I); and abnormally large mitochondria (E, J) (see arrowheads for selected abnormal mitochondria). Disruption of the normal alignment of mitochondria (mt) with myofibrils (mf) is shown in representative newborn control (wildtype) versus TAZKD cardiomyocytes (K, L). Scale bars: 500 nm. TAZKD indicates *tafazzin* knockdown.

**Figure 5.**
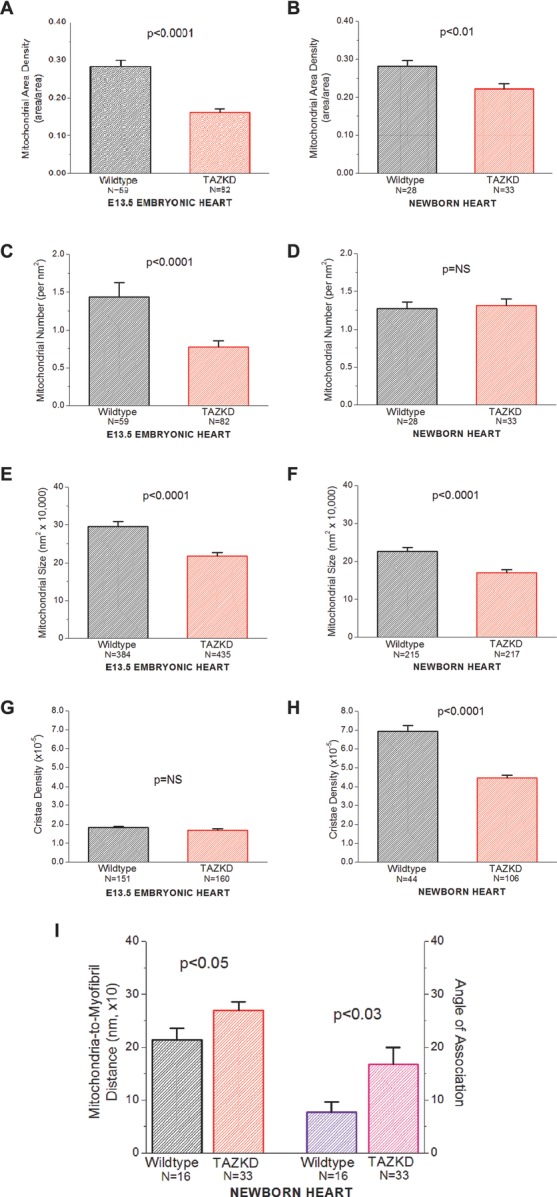
Morphometric analysis of mitochondria reveals distinct abnormalities in TAZKD mice. Mitochondrial structures were analyzed from electron micrographs taken of E13.5 embryonic hearts (A, C, E, G) and newborn hearts (B, D, F, H, I). TAZKD cardiomyocytes demonstrate statistically significant decreases in mitochondrial area density (embryonic, newborn), mitochondrial number per unit area (embryos), mitochondrial size (embryonic, newborn), and cristae density (newborns). Newborn myocardium exhibits disruptions in the spatial relations between mitochondria and myofibrils, as indicated by increased distances and a less-parallel geometry between mitochondria and myofibrils (I) (NB: Embryonic myocardium does not exhibit myofibrillar units well-developed enough for analysis). In A, B, C, F, and I, the *N* shown are the number of fields used for data analysis; in E, F, G, and H, *N* is the number of mitochondria analyzed. TAZKD indicates *tafazzin* knockdown.

Quantitative analysis of electron micrographs showed a spectrum of abnormalities in mitochondrial ultrastructure and sarcomeric organization, including vacuolated cristae, a reduction in total mitochondrial area density, a reduction in mitochondrial size, and lower cristae density. Also, newborn TAZKD mice demonstrated a disruption of the normal parallel orientation between mitochondria and sarcomeres (Figures 4 and 5). Overall, these abnormalities indicate abnormal mitochondria and are consistent with delayed cardiomyocyte differentiation.^21^

### TAZKD Mouse Hearts Exhibit Distinct Functional Abnormalities at the Embryonic Stage

We studied whether cardiomyopathy could explain the prenatal and perinatal deaths. As reflected by normal cardiac dimensions and fractional area change, systolic function was preserved in both TAZKD mouse embryos and newborn pups (Tables 2 and 3). However, peak dorsal aortic velocity was significantly lower in the TAZKD than control wildtype embryos, which might suggest subtle systolic dysfunction, and this was even lower in more severely affected TAZKD embryos with an “a-kick” (see later). Heart rates, atrioventricular conduction (PR interval), and umbilical arterial blood flows (a surrogate of placental health) were not significantly different between TAZKD and controls, although heart rates in TAZKD embryos trended lower in more severely affected embryos and as gestation progressed (Table 2).

**Table 2.** In Vivo UBM-Derived Embryonic Hemodynamic Measurements

	Embryonic Stage			
Functional Parameter	E12.5	E13.5	E13.5 (TAZKD+a-kick)	E14.5
*N* (Total Numbers With Usable Data; Not All Measurements Could Be Obtained in All Embryos)				

Wildtype	8	16	16	6

TAZKD	5	16	3	3

Heart Rate, beats/min				

Wildtype	132±13	136±7	136±7	126±6

TAZKD	140±24	143±13	101±14	109±8

End-Diastolic Area, mm^2^ (From 4-Chamber View)				

Wildtype	1.765±0.101	1.969±0.057	1.969±0.057	2.503±0.107

TAZKD	1.695±0.054	1.832±0.072	1.720±0.315	2.501±0.114

Fractional Area Change, %				

Wildtype	36.2±1.8	42.1±1.7	42.1±1.7	44.9±2.9

TAZKD	45.2±1.3*	45.5±1.3	46.0±3.3	52.5±1.2

PR Interval, ms				

Wildtype	98±12	119±6	119±6	120±6

TAZKD	97±24	121±7	151±12	96

Isovolumic Contraction Time, ms				

Wildtype	27±8	40±4	40±4	33±4

TAZKD	43±6	51±5	59±11	21

Isovolumic Relaxation Time, ms				

Wildtype	61±18	53±8	53±8	44±1

TAZKD	45±5	42±9	23±19	20

DA Peak Velocity, mm/s				

Wildtype	100±13	103±8	103±8	126±11

TAZKD	85±22	78±8*	59±12*	115±29

Atrial “kick” Wave, *N* (%)				

Wildtype	0	0	0	0

TAZKD	1 (20%)	3 (19%)	(3)	3 (100%)

DA ET, ms				

Wildtype	174±12	172±6	172±6	186±6

TAZKD	156±14	158±9	187±6	196±9

UA Peak Velocity, mm/s				

Wildtype	75±9	75±5	75±5	103±9

TAZKD	67±12	67±6	60±12*	91±5

UV Peak Velocity, mm/s				

Wildtype	45±7	48±5	48±5	68±7

TAZKD	39±4	37±4	27±5*	43±2*

UV Trough Velocity, mm/s				

Wildtype	15±3	17±2	17±2	21±4

TAZKD	6±3	11±2*	4±2†	5±3*

UV Peak/Trough Ratio				

Wildtype	0.34±0.03	0.34±0.02	0.34±0.02	0.31±0.03

TAZKD	0.16±0.07*	0.29±0.04	0.20±0.12	0.13±0.08

Data are expressed as mean±SEM.

DA indicates dorsal aorta; ET, ejection time; TAZKD, *tafazzin* knockdown; UA, umbilical artery; UV, umbilical vein.

“E13.5 (TAZKD + a-kick)” represents the statistical comparisons between E13.5 wildtype and only those TAZKD embryos exhibiting an abnormal dorsal aortic “a-kick” (*N*=3). Notably, of the E14.5 litters imaged and assayed, all TAZKD embryos either were dead or exhibited an abnormal “atrial kick’ in the dorsal aorta. Values without SEM represent available data from one embryo only. TAZKD compared with WT.

* *P*<0.05;

† *P*<0.01.

**Table 3.** In Vivo UBM-Derived Newborn Hemodynamic Measurements

	Newborn	
Functional Parameter	Wildtype	TAZKD
*N*	16	9

Weight, g	1.15±0.05	1.01±0.04*

Heart rate, beats/min	260±13	244±3

PR interval, ms	76±3	82±3

Isovolumic contraction time, ms	24±5	31±11

Isovolumic relaxation time, ms	47±1	50±6

Diastolic septal thickness, mm	0.27±0.01	0.27±0.01

Diastolic LV dimension, mm	1.34±0.03	1.31±0.04

Diastolic LV posterior wall, mm	0.26±0.01	0.26±0.01

Systolic septal thickness, mm	0.39±0.01	0.39±0.01

Systolic LV dimension, mm	0.92±0.03	0.93±0.03

Systolic LV posterior wall, mm	0.40±0.03	0.35±0.01*

LV shortening fraction,%	31±2	29±1

LV end-diastolic area, mm^2^	1.413±0.070	1.375±0.058

LV fractional area change,%	50.8±1.4	49.4±1.1

Data are expressed as means±SEM.

LV indicated left ventricular; TAZKD, *tafazzin* knockdown.

NB: Because of technical challenges, only a limited subset of data could be obtained on newborn pups. It should be noted that newborn pups are not anesthetized during imaging. There were no significant differences between wildtype and TAZKD newborn echo functional parameters; although systolic LV posterior wall achieved statistical significance (*P<0.05), the large number of comparisons in this table and the absence of other differences in cardiac morphometry and function cast doubt as to the relevance of this finding.

* Weight was significantly lower in the TAZKD newborn pups (*P*<0.05).

**Figure 6.**
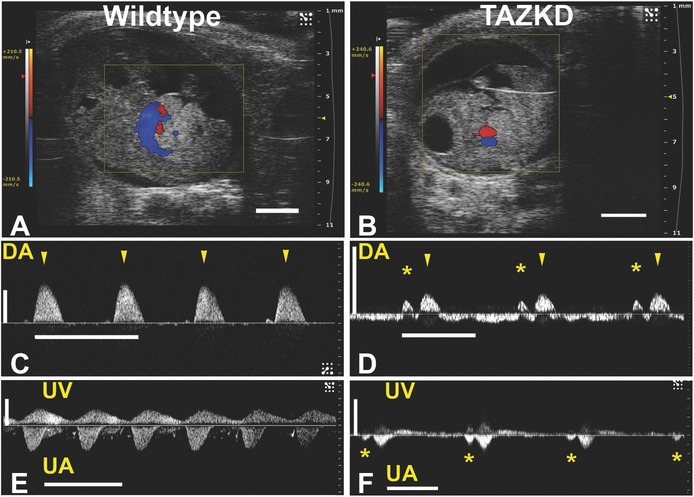
Cardiac dysfunction is evident in some TAZKD embryonic mice. Some TAZKD embryos exhibit severe diastolic dysfunction with a unique “atrial kick” in the dorsal aorta, at E12.5, 13.5, and 14.5, as revealed by spectral Doppler analysis of dorsal aortic blood flow; images are of an E13.5 wildtype embryo (A, C, E) and TAZKD embryo (B, D, F). Sagittal views of control wildtype (A) and TAZKD (B) embryos with superimposed color Doppler map show blood flow, but no obvious abnormalities such as hydrops (scale bars = 2 mm). Normal all-antegrade dorsal aortic (DA) blood flow is seen in the wildtype embryo (C), with peak aortic pulsations indicated by the arrowheads. Blood flow in the TAZKD dorsal aorta (D) shows an “atrial kick” (asterisks) occurring just before the peak aortic pulsations (arrowheads). In umbilical artery (UA) and umbilical vein (UV), the TAZKD embryo (F) shows propagation of the atrial kick even into the umbilical artery (asterisks), not evident in the wildtype embryo (E). Also note peak aortic velocities in TAZKD embryos (D, F) are lower than in WT littermates (C, E). Time (horizontal) scale bars for panels C to F = 0.5 s; velocity (vertical) scale bar = 100 mm/s. TAZKD indicates *tafazzin* knockdown.

Notably, flow in the dorsal aorta of several TAZKD embryos showed a distinct peak immediately preceding the normal antegrade flow envelope; this was shown to originate from atrial contraction by Doppler interrogation of the outflow tract (Figure 6). The presence of an atrial systolic kick in the aorta indirectly suggests ventricular diastolic dysfunction, with a left ventricle so stiff that the atrial contraction, exceeding aortic pressure, is transmitted into the aorta. Although we did not validate the Doppler findings by other means, this contention is supported by the umbilical venous flow patterns, which showed lower peak and trough velocities, suggesting increased “downstream” impedance and therefore diastolic dysfunction in the heart. Importantly, diastolic dysfunction preceded overt systolic dysfunction, but there was no evidence of progressive ventricular dilatation. An A-wave in a downstream artery is a well-known echocardiographic indicator of abnormally elevated ventricular stiffness,^25^ and patients with myocardial noncompaction almost always exhibit diastolic dysfunction.^26^ That atrial pressures can exceed aortic pressures is plausible in the mouse embryo, because intracardiac and intraarterial pressures are very low.^22^ Diastolic dysfunction with preserved systolic function has been demonstrated in the friend of GATA-2 (FOG-2) null mouse that also develops noncompaction.^22^ Although another measure of diastolic function, isovolumic relaxation time, appeared normal in TAZKD mice, it should be noted that the cardiac valves are not yet completely mature in mouse embryos and are unlikely to behave like the mature, thin, and delicate valves of an adult heart that lead to “normal” isovolumic time intervals.^27^

While diastolic dysfunction may lead to pre-/perinatal lethality in this model, we cannot exclude a malignant arrhythmia leading to sudden cardiac death, a well-established feature of Barth syndrome.^3^ Indeed, we speculate the normal newborn TAZKD echo data (Table 3) may represent a survival bias, and therefore they died not from cardiac dysfunction, but even more likely from malignant arrhythmia.

### TAZKD Mouse Hearts Develop Myocardial Hypertrabeculation-Noncompaction Cardiomyopathy Because of Aberrations in Early (<E13.5) Embryogenesis

**Figure 7.**
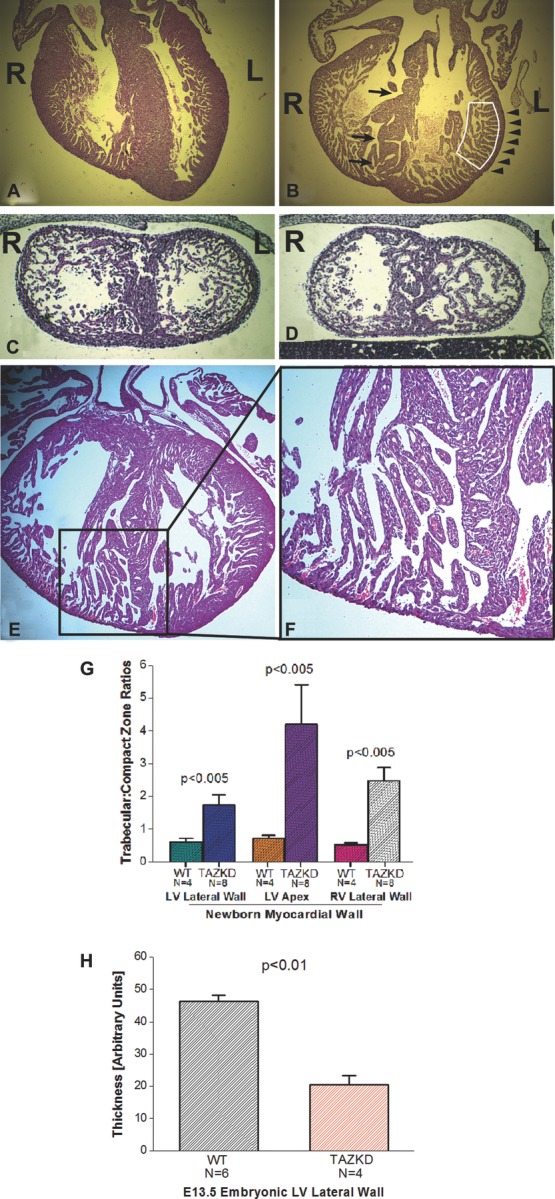
TAZKD mice exhibit thinner myocardium with prominent trabeculations suggestive of myocardial noncompaction. “4-chamber” views of newborn WT control (A) and TAZKD (B) hearts show deeper trabecular recesses and more abundant trabeculae (boxed area), thinner myocardium (arrowheads), and a less well-developed and more perforate interventricular septum in TAZKD hearts (arrows) (R = right, L = left). Coronal sections of E13.5 embryonic WT control heart (C) and TAZKD (D) hearts also demonstrate a thinner myocardium with more prominent trabeculae that occupy more of the left ventricular lumen in TAZKD hearts; again, the septum appears more porous. *Tafazzin* knockdown in these mice (A to D) was induced from start of gestation. Induction of TAZKD starting at E10.5 (E) recapitulates the cardiomyopathic phenotype, as represented by this newborn TAZKD heart (enlargement of apex in [F]). TAZKD newborn pups show a significantly higher degree of hypertrabeculation-noncompaction and myocardial thinning in all areas, but most notably the LV apex (G). In E13.5 TAZKD littermate embryos (H), significant myocardial thinning is also evident. Because this embryonic stage still exhibits a normally prominent trabecular myocardium, quantitation of trabecular-compact zone myocardium thicknesses was not performed. TAZKD indicates *tafazzin* knockdown; WT, wildtype.

To further understand the cause of the diastolic dysfunction, we examined histological sections of embryonic and newborn mouse hearts. TAZKD mice showed myocardial hypertrabeculation with noncompaction throughout development. Hypertrabeculation-noncompaction was also associated with defects in ventricular septation (Figure 7). There was no evidence of myocardial fibrosis (data not shown). Uninduced control mice (wildtype and TAZ transgenic) showed normal survival and heart structure (data not shown); therefore, our findings could not be attributed to the presence of the transgene itself.

**Figure 8.**
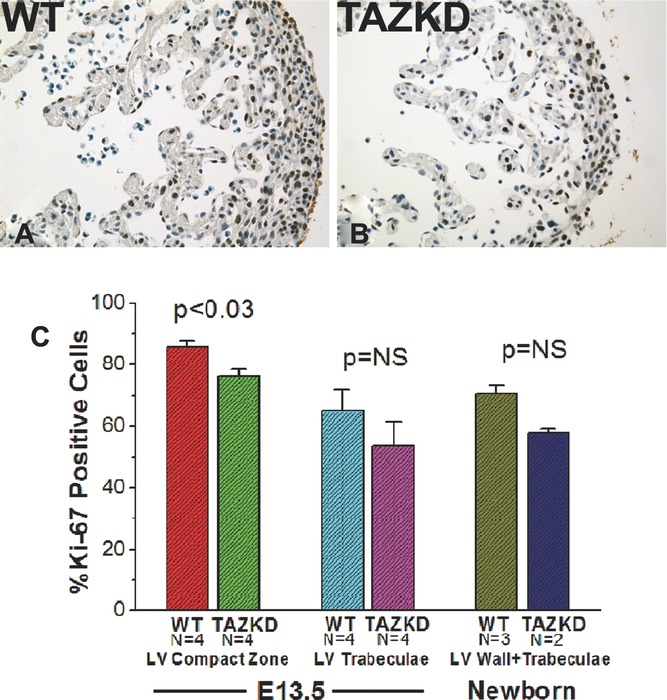
Myocardial noncompaction cardiomyopathy is accompanied by abnormal cellular proliferation. Cellular proliferation immunostaining shows Ki-67-positive nuclei (proliferating cells) in brown color (A, E13.5 wildtype [WT] embryo; B, TAZKD embryo). TAZKD mice exhibit significantly lower cellular proliferation rates (C). Because in normal embryonic development, trabecular and compact zone myocardium exhibit differential proliferation rates, we measured cellular proliferation separately in E13.5 trabecular and compact zones (C, left-hand and middle columns); TAZKD embryos exhibit significantly lower proliferation rates in the compact zone, but the differences were not significant in the trabecular zone. TAZKD indicates *tafazzin* knockdown.

Development of trabeculations followed by myocardial compaction are steps in myocardial patterning critical to embryonic heart functioning and development of the cardiac conduction system. The sequence in the mouse occurs specifically from E10.5 to E14.5.^28,29^ To determine the approximate developmental window of the origins of cardiomyopathy in our model, we varied the timing of doxycycline induction. First, we determined that *taz* mRNA knockdown reaches steady state (>70%) within 72 hours of doxycycline induction. Then, we demonstrated that induction with doxycycline at the start of gestation, at E7.5, and at E10.5 recapitulated the hypertrabeculation-noncompaction cardiomyopathy (Figure 8), whereas induction at a later stage (E13.5 and E14.5) failed to result in either early lethality or a structural cardiomyopathy. Therefore, the developmental cardiomyopathy hinges specifically on tafazzin dysfunction in the embryonic period following E10.5 but not extending past E13.5, suggesting tafazzin plays a critical role in heart development during this window.

Molecular mechanisms underlying myocardial hypertrabeculation-noncompaction are unknown, but several mouse models point to cell cycling alterations.^30–34^ We therefore determined differences in cellular proliferation and apoptosis in E13.5 and newborn hearts. There were no differences in apoptosis at any stage (data not shown), consistent with other models of hypertrabeculation-noncompaction.^34^ Cellular proliferation was decreased in TAZKD mice; furthermore, trabecular myocardium showed lower proliferation rates than compact myocardium (Figure 8). Taken together, our data indicate myocardial hypertrabeculation-noncompaction is associated with abnormal cell cycling starting in the broad developmental window when the myocardium should be compacting.

## Discussion

These data are the first to demonstrate a developmental cardiomyopathy in this murine model of tafazzin knockdown. Notably, myocardial hypertrabeculation with noncompaction is consistently seen in this model. The constellation of abnormalities highlights this TAZKD mouse as a promising model to study the human Barth syndrome. Importantly, the data point to important aspects of mitochondrial functioning in the development of myocardial patterning and tissue architecture.

Prior reports of this model have demonstrated similar biochemical and mitochondrial abnormalities, but only in adult mice; cardiomyopathy did not develop until well into adulthood.^11,12^ We believe our model was induced by a higher-than-previously reported dosing protocol of doxycycline induction. Seibler et al^13^ showed the dose of doxycycline influenced especially the speed with which the knockdown achieved steady-state levels. Prior studies^11,12^ did not demonstrate tafazzin knockdown during embryogenesis, so their data cannot be compared directly with ours. When we carried our doxycycline induction protocol in young postnatal mice, tafazzin was knocked down to 3.8±0.68% of control levels in heart, lower than Soustek et al^12^ but similar to Acehan et al^11^; we speculate the kinetics of induction were also faster because of our higher dosage, generating the embryonic phenotype.

In our model, myocardial hypertrabeculation-noncompaction developed during a critical developmental window, consistent with what we know about the normal compaction process.^28,29^ Therefore, the cardiomyopathy observed by others^11,12^ appears to represent a different process than the developmental cardiomyopathy reported here. We acknowledge that because none of our mice survived beyond the early postnatal period, the model does not perfectly mirror human Barth syndrome. Nevertheless, the hallmark phenotype of hypertrabeculation-noncompaction is more similar to human Barth syndrome than reported previously.^11,12^

We cannot entirely exclude off-target effects of this transgenic knockdown strategy, similar to previous reports,^11,12^ because only one shRNA knockdown mouse line was available. Nevertheless, recapitulating the developmental cardiomyopathy seen in Barth syndrome, and the combination of findings—molecular, biochemical, ultrastructural, and functional—make off-target effects of our knockdown strategy unlikely. Nevertheless, additional problems, such as abnormal ventricular septation and myocardial proliferation, may be due to off-target effects. Furthermore, this model relies on induction with doxycycline. Doxycycline may be associated with placental anomalies and fetal loss, especially when introduced into food but seemingly not water.^35^ However, we detected no gross losses in our wildtype mice. Doxycycline may attenuate the pathophysiology of cardiac failure through matrix metalloproteinase inhibitor mechanisms,^36^ although the very presence of hypertrabeculation-noncompaction with early lethality in our model argues against a significant protective effect of doxycycline.

### Myocardial Hypertrabeculation-Noncompaction

Myocardial noncompaction is a poorly understood developmental cardiomyopathy,^5,37^ now recognized as a distinct cardiomyopathy.^38^ Patients with Barth syndrome present as early as fetal life with cardiomyopathy,^4^ and conversely, noncompacted ventricular myocardium also presents with a clinical spectrum starting in fetal and neonatal life.^39^ Ventricular septal defects are often associated with noncompaction, both in human beings and in animal models.^5,40^ Mitochondrial disorders are strongly associated with myocardial noncompaction.^5,38,41,42^ Nevertheless, not all mitochondrial disorders lead to hypertrabeculation-noncompaction, and therefore, it seems likely only certain aspects of mitochondrial functioning contribute to this condition.

The early diastolic dysfunction is notable. Because there was no fibrosis or abnormal myocardial hypertrophy, we speculate alterations in cardiolipin involve abnormal myocardial physiology, such as altered calcium homeostasis (see later), that precede the overt hypertrabeculation-noncompaction. Interestingly, no severe systolic dysfunction was observed; but then, the model reported by Acehan et al^11^ and Soustek et al^12^ exhibited only a mild cardiomyopathy in adulthood without overt heart failure. We speculate we did not observe the “natural history” of this mouse, perhaps because of a lethal arrhythmia or cannibalization of unhealthy-looking pups by the mother; also, while human infants with severe cardiomyopathy are treated, mice are not.

Hypertrabeculation-noncompaction may be the result of dysregulated cellular proliferation in the myocardium,^5,30,37,43^ although the precise molecular mechanisms remain elusive. Preceding compaction, formation of normal trabecular myocardium plays a critical role in normal myocardial development, and depends on Notch signaling, while exhibiting differential cardiomyocyte proliferation in different layers of the developing heart.^44^ Our data suggest a dysregulation of this differential cardiomyocyte proliferation: specifically, a thin compact zone exiting earlier from the cell cycle while the trabecular zone continues to proliferate at a more normal level may explain our hypertrabeculation phenotype. We did not distinguish different cell types for this experiment, and therefore cannot exclude nonmyocytes as the main contributor to the decreases in cell proliferation; nonmyocytes may exert as-yet unknown influences on myocardial patterning. These questions will be investigated in ongoing studies.

### The Role of Mitochondria in Heart Development

The role of mitochondria in the development of a structural cardiomyopathy is intriguing. Emerging evidence suggests mitochondrial disorders account for a substantial proportion of not simply cardiomyopathies, but specifically myocardial hypertrabeculation-noncompaction and other abnormalities in the development of the heart and other organs.^45,46^

Although investigators have focused logically on bioenergetics, more recent evidence also indicates a strong interrelationship between mitochondria, calcium homeostasis via the sarcoplasmic reticulum and sarcomeric organization.^47^ We now provide additional evidence that altered cardiolipin biology leads to a disruption of the normal spatial relation between mitochondria and sarcomeres in the newborn heart. We do not know at present how this disruption will mechanistically contribute to the developmental cardiomyopathy, but speculate excitation-contraction coupling may well be damaged. We acknowledge other mechanisms may also be intertwined, including the possible role of reactive oxygen species and bioenergetics of the developing heart.^48^

### Conclusions and Future Directions

This is the first report of a murine model of tafazzin knockdown exhibiting a developmental cardiomyopathy that closely mimics the human Barth syndrome. Taken together, our data indicate certain aspects of mitochondrial functioning are necessary for proper myocardial patterning, and provide additional evidence of a causative role of mitochondrial dysfunction in the pathogenesis of myocardial hypertrabeculation-noncompaction. Although our data here offer no specific mechanistic insights, aspects of mitochondrial function that are candidates for study include the bioenergetic pathways and calcium homeostasis pathways, as well as the roles of reactive oxygen species.^44,45,47^
